# An inclusive assessment of apoptosis mechanisms in *Leishmania* species: A narrative literature review

**DOI:** 10.1016/j.crpvbd.2025.100260

**Published:** 2025-04-17

**Authors:** Soheil Sadr, Iraj Sharifi, Solmaz Morovati, Helia Sepahvand, Shakiba Nazemian, Mehdi Bamorovat, Zahra Rezaeian, Baharak Akhtardanesh

**Affiliations:** a Department of Pathobiology, Faculty of Veterinary Medicine, Ferdowsi University of Mashhad, Mashhad, Iran; b Leishmaniasis Research Center, Kerman University of Medical Sciences, Kerman, Iran; c Division of Biotechnology, Department of Pathobiology, School of Veterinary Medicine, Shiraz University, Iran; d University of Tehran, Tehran, Iran; e Department of Clinical Sciences, Faculty of Veterinary Medicine, Shahid Bahonar University of Kerman, Kerman, Iran

**Keywords:** Leishmaniasis, Apoptosis, Programmed cell death, Parasite-host interactions, Immune evasion, Drug discovery

## Abstract

Leishmaniasis, the most neglected tropical disease caused by protozoan parasites of the genus *Leishmania*, poses a substantial global health concern. The present review provides an in-depth overview of current findings on apoptosis and cell death mechanisms in leishmaniasis, integrating current advancements and key components. It explores the intricate interaction between *Leishmania* spp. and host cell apoptosis, a crucial basis of disease outcome. *Leishmania* spp. and host cell death pathways interplay is highly complex and multi-layered, and the current review discusses how *Leishmania* parasites manipulate host cell apoptotic signal transduction to establish and sustain infection. This includes the subversion of intrinsic and extrinsic apoptotic signaling, the modulation of pro- and anti-apoptotic proteins, and managing host cell death machinery for their survival and pathogenesis. Moreover, the present review explores the emerging evidence of apoptosis in *Leishmania* parasites. This fascinating phenomenon, while less widely studied, recommends immense therapeutic potential in targeting parasite-persistent mechanisms. Finally, we critically analyze the challenges and future directions in this field, emphasizing the need for a deeper understanding of the merits and mediator molecular mechanisms underlying *Leishmania*-induced apoptosis and its implications for novel therapeutic strategies against this debilitating disease.

## Highlights

• *Leishmania* spp. exhibit processes similar to eukaryotic apoptosis but lack classical caspases.
• Reactive oxygen species play a key role in inducing programmed cell death in *Leishmania.*
• Changes in pH and oxidative stress can trigger apoptosis in *Leishmania.*
• Genes such as metacaspase and endonuclease G are essential in *Leishmania* cell death.
• Studying apoptosis in *Leishmania* can help identify new drug targets for treating leishmaniasis.

## Graphical abstract

**Figure:**
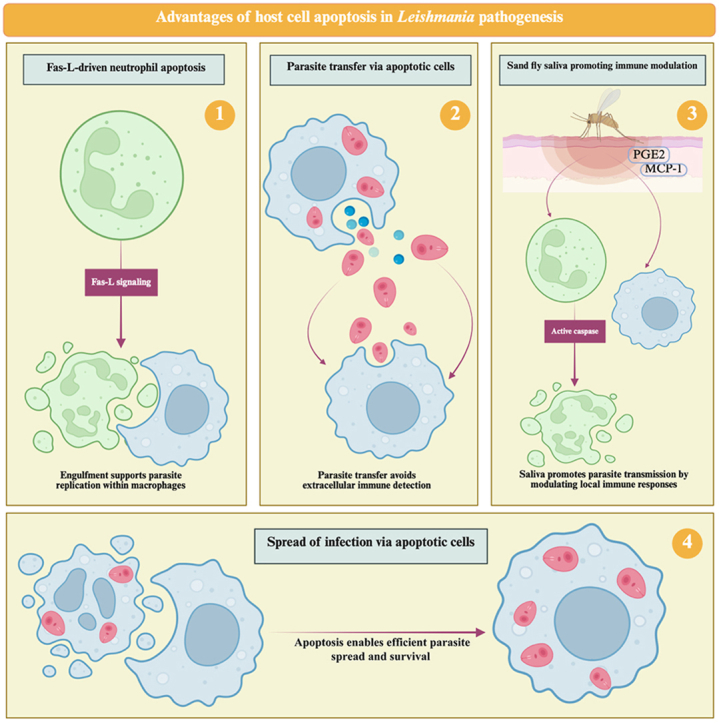

## 1 Introduction

Leishmaniasis is an infection caused by over 20 species of *Leishmania*, part of the family Trypanosomatidae (Torres-Guerrero et al., 2017; Espinosa et al., 2018). These flagellated parasites lead to conditions with varying severity, from skin lesions to systemic complications. The disease presents in three classical forms: visceral (Kala-azar, VL), and localized cutaneous leishmaniasis (CL) (DaMata et al., 2015). These manifestations depend on factors such as the parasite species, the infection site, the host immune response, the spatial distribution, and genetic predisposition (Kedzierski and Evans, 2014).

The World Health Organization classifies leishmaniasis as a significant, overlooked tropical illness, predominantly impacting low-income and impoverished populations globally. There are currently about 12 million people affected, and 0.7–1.2 million new cases are reported each year, over 80 % of which are CL (WHO, 2014). The figure projects only a small fraction of people infected with *Leishmania* spp. Over 350 million people across 98 countries and three territories are at risk (Postigo, 2010; Alvar et al., 2012; WHO, 2016; Wamai et al., 2020). Leishmaniasis is associated with population movement, malnutrition, poor housing conditions, deficient immune response, chronic diseases, poor treatment adherence, disasters, parasite resistance, climate change, and man-made and environmental alterations (Aflatoonian et al., 2014, 2019; Bamorovat et al., 2018, 2019, 2021a, b, 2023, 2024a, b; Ilaghi et al., 2021; Mohebali et al., 2023; Sharifi et al., 2023). The disease presents a significant health challenge due to the lack of a vaccine, inadequate treatments, high costs, adverse effects, the need for parenteral administration (which limits access in developing regions), and the growing issue of drug-resistant strains (Keyhani et al., 2021; Salarkia et al., 2023; Sadr et al., 2024; Shahsavari et al., 2024).

During a blood meal, female sand flies inject infectious promastigotes into the skin of vertebrate hosts, causing infection. Inside the sand fly, the parasites multiply as flagellated promastigotes, transitioning from procyclic forms in the midgut to virulent metacyclic forms in the proboscis (Bates, 2008). Once in the dermis, macrophages pick promastigotes and convert them into pathogenic amastigotes, which reside within acidic phagolysosomes. These amastigotes exit and rupture, harboring macrophages to infect adjacent cells. Amastigotes transform into promastigotes when a sand fly ingests them, thus completing the life cycle (Dostálová and Volf, 2012).

Apoptosis, as a process of programmed cell death, plays an important role in regulating cell population and survival of living organisms. In protozoan parasites such as *Leishmania* spp., which are the causative agents of leishmaniasis in humans and animals, apoptosis is not only effective in parasite survival in variable host environments but also plays a key role in modulating host immune responses (Shaha, 2006; Elmahallawy and Alkhaldi, 2021). Unlike complex eukaryotic cells, apoptotic pathways in *Leishmania* spp. are unusual and adapted to the parasite’s environmental conditions, which can be exploited as unique therapeutic targets (Veras and Bezerra de Menezes, 2016; dos Santos Meira and Gedamu, 2019). Understanding the precise mechanism of apoptosis in *Leishmania* spp. can provide fundamental insights into their survival and pathogenicity and play a crucial role in developing novel therapeutic approaches to control disease (Cecílio et al., 2014; Clos et al., 2022).

This review investigates the mechanism of apoptosis in *Leishmania* spp. and the molecular and cellular processes associated with apoptosis in these protozoan parasites. Additionally, it aims to elucidate the role of apoptosis in parasite survival, regulation of cell populations, and its impact on *Leishmania* spp. pathogenicity. Moreover, by reviewing the existing research, the authors seek to identify key molecules and pathways involved in this process, which could help develop new therapeutic strategies for controlling leishmaniasis.

## 2 Search strategies

This review generally emphasizes the studies published from 2015 to 2024 on the mechanism of apoptosis in *Leishmania* spp. and the molecular and cellular processes associated with apoptosis in these protozoan parasites. A wide-ranging literature search was conducted in Google Scholar, PubMed, and MEDLINE databases. Literature review, research, and other different types of articles and books were investigated using the following keywords: “apoptosis”, “leishmaniasis”, “cutaneous leishmaniasis”, “visceral leishmaniasis”, “programmed cell death”, “parasite-host interactions”, “treatment”, “immune evasion”, “drug discovery”, “treatment failure”, “*Leishmania*”, and “proteins involved in apoptosis”. Exclusion and inclusion criteria, such as date, type, journal quality, language, exposure interest, and reported consequences, were applied for specific concepts in the study sources. The selected sources were assessed and reviewed by all the authors according to the research criteria and questions, and lastly, the preferred results were extracted.

## 3 Life cycle

**Fig. 1:**
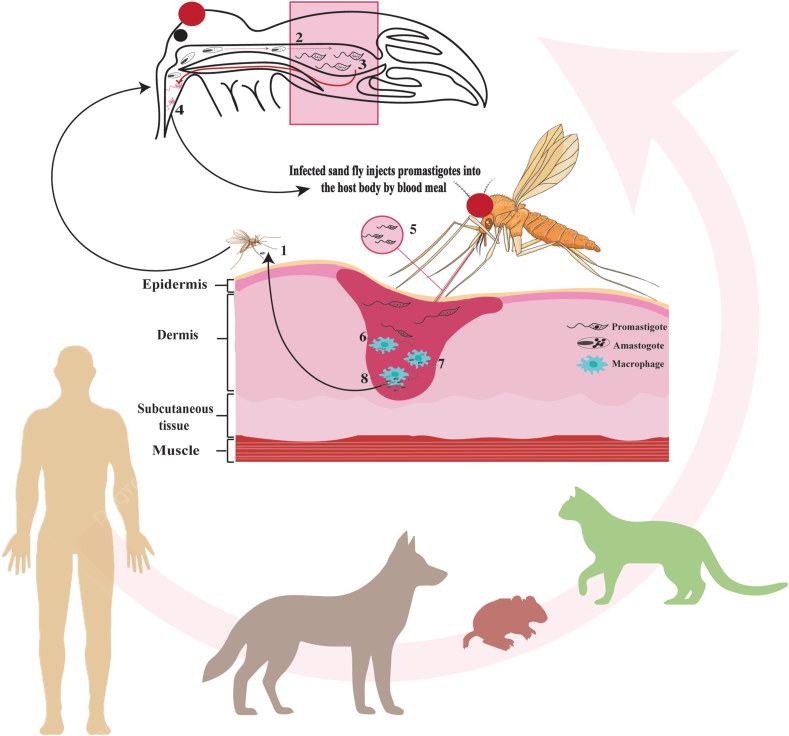
The life cycle of *Leishmania* spp. and the interaction with sand flies and humans, canines, felines, and murines as hosts. (**1**) Amastigote is being ingested by the sand fly during blood-feeding. (**2**) The amastigote is transformed into promastigote in sand fly midgut. (**3**) In the midgut, promastigotes replicate. (**4**) Promastigotes migrate to the pharynx. (**5**) Infected sand fly injects promastigotes into humans or other hosts through blood meals. (**6**) Promastigotes are engulfed by macrophages when they enter the host skin. (**7**) Inside macrophages, promastigotes transform into amastigotes. (**8**) In phagolysosomes, amastigotes multiply and are released to infect new cells by rupturing macrophages.

The life cycle of *Leishmania* spp. involves sand flies of the genera *Phlebotomus* or *Lutzomyia*, acting as both intermediate hosts and vectors. The female sand fly is the primary transmitter of the disease (Moriconi et al., 2017). When an infected host’s blood is ingested (Fig. 1(1)), the amastigotes present in the blood transform into promastigotes within the sand fly’s midgut (Fig. 1(2-3)). These promastigotes proliferate, migrate to the foregut, and are subsequently transmitted to a new host during the fly’s next blood meal (Fig. 1(4-5)). The life cycle spans approximately ten days (Gossage et al., 2003). Upon biting an uninfected host, the sand fly injects the stationary phase promastigotes into the skin tissue (Rogers, 2012; Inbar et al., 2017; Bamorovat et al., 2024a). These promastigotes are then phagocytized by macrophages, where they differentiate into amastigotes and replicate intracellularly by longitudinal binary fission (Fig. 1(6-7)) (KarimAlqaraghli, 2024). The proliferation of amastigotes eventually leads to macrophage rupture, facilitating the infection of adjacent cells (Fig. 1(8)). The life cycle is completed when another sand fly feeds on the infected host, ingesting amastigotes and further perpetuating transmission (Fig. 1(1)) (Bates, 2007; Pimenta et al., 2018; Cecílio et al., 2022).

Promastigotes are coated with glycocalyx, which safeguards them from host hydrolases and serum components within the insect’s digestive tract (Tom et al., 2024). Once injected into mammalian skin, metacyclic promastigotes demonstrate resistance to serum factors and are engulfed by macrophages, initiating infection (Fig. 1). Many lines of research indicate that *Leishmania* parasites reside and replicate within phagolysosomal compartments (McConville et al., 2007; Séguin and Descoteaux, 2016; Matte et al., 2021). Amastigotes, which exhibit high resistance to environmental stresses, contain hydrolytic enzymes that interfere with endosomal and lysosomal functions. They withstand the acidic pH (4.7–5.2) of the vacuole due to the activity of H^+^-ATPase (Lazar and Abass, 2020). Furthermore, amastigotes endure oxidative and nitrosative stress caused by reactive oxygen species (ROS) and nitric oxide synthase (NOS) activity within macrophages (Naderer and McConville, 2008; Van Assche et al., 2011; Duque and Descoteaux, 2015; Podinovskaia and Descoteaux, 2015). Furthermore, metabolic processes such as mitochondrial respiration contribute to oxidative stress (Kaye and Scott, 2011). The deletion or impairment of any oxidative defense mechanism in *Leishmania* compromises its virulence.

## 4 Apoptosis

**Fig. 2:**
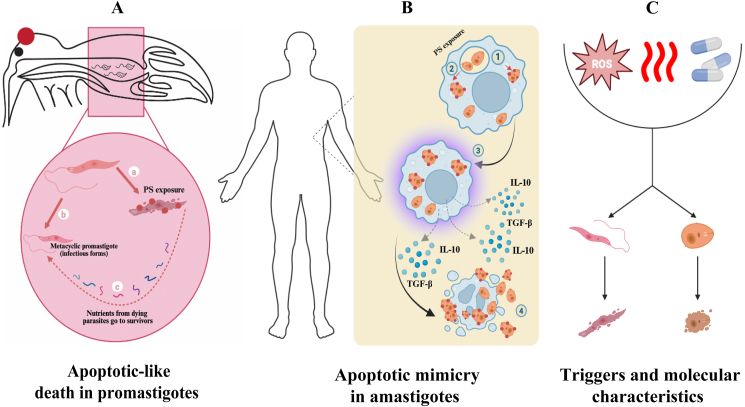
Cell death mechanisms in *Leishmania* spp. **A** Apoptotic-like death in promastigotes: Phosphatidylserine (PS) exposure transforms the promastigotes into apoptotic forms in the gut of sandflies (**a**). Dead promastigotes are shown to disintegrate, and nutrients are saved for infectious metacyclic forms (**b**). PS exposure and Annexin-V binding are markers of apoptosis-like death, but regulated cell death (RCD) ensures the survival of the fittest (**c**). **B** Apoptotic mimicry in amastigotes: PS-exposed amastigotes engulfed by macrophages (**1**, **2**); apoptotic mimicry suppresses the immune system by secretion of anti-inflammatory cytokines like IL-10 and TGF-β (**3**); reducing the immune response aids parasite survival (**4**). **C** Triggers and molecular characteristics: radical oxygen species (ROS), drugs such as miltefosine, and heat shock can cause apoptosis in parasites. Created in BioRender (https://BioRender.com/m85k410).

Apoptosis is a unique form of programmed cell death crucial for embryonic development, maintaining tissue balance, and controlling diseases in multicellular organisms (Chen et al., 2024). Apoptosis describes a process characterized by specific morphological changes: cell rounding, pseudopod retraction, shrinkage, chromatin condensation, nuclear fragmentation, and minimal alterations in cytoplasmic organelles (Solano-Gálvez et al., 2018). The plasma membrane remains intact while undergoing blebbing, eventually forming apoptotic bodies. Carried out in a living organism, these bodies are typically engulfed by surrounding cells, completing the process of apoptosis (Fig. 2) (Kroemer et al., 2009).

The classical pathways involved in apoptosis include the intrinsic and extrinsic pathways. Both pathways are interconnected and can influence each other during the cell death process (Jin and El-Deiry, 2005). The extrinsic pathway is begun by extracellular signals, such as those from neighboring cells or immune responses that trigger death receptors on the cell surface (Bedoui et al., 2020). In contrast, the intrinsic pathway is initiated by intracellular signals, such as oxidative stress, DNA damage, or other disruptions within the cell (Nair et al., 2014). This pathway is regulated by the Bcl-2 family of proteins, which include pro-apoptotic members (e.g. Bak and Bax) and anti-apoptotic members (e.g. Bcl-xL and Bcl-2) (Green and Llambi, 2015; Redza-Dutordoir and Averill-Bates, 2016; Khosravi et al., 2022; Mosallanejad et al., 2022). These proteins regulate mitochondrial outer membrane permeabilization (MOMP), a crucial step in the process. MOMP leads to the release of molecules like cytochrome *c* from mitochondria into the cytoplasm. Released cytochrome *c* forms a complex with other proteins to activate caspases (Fadeel and Orrenius, 2005; Kist and Vucic, 2021). Caspases are responsible for the controlled dismantling of the cell, ensuring it dies without causing inflammation or damage to surrounding tissues (Elmore, 2007).

## 5 Cell death mechanisms in *Leishmania* spp.

*Leishmania* spp. can exhibit cell death mechanisms resembling apoptosis in higher eukaryotes (Basmaciyan and Casanova, 2019). These mechanisms include apoptotic-like death (Fig. 2A) and apoptotic mimicry (Fig. 2B). While apoptotic death refers to a programmed type of cell death, apoptotic mimicry involves imitating the features of apoptotic cells, often through the exposure of phosphatidylserine (PS) on the cell surface (Fig. 2A,B) (El-Hani et al., 2012; Wanderley et al., 2020). In murine infections caused by *Leishmania* spp., apoptosis has been observed in promastigotes, whereas amastigotes display features of apoptotic mimicry (de Freitas Balanco et al., 2001; Wanderley et al., 2006; Wanderley and Barcinski, 2010; El-Hani et al., 2012). True apoptosis is debated because *Leishmania* spp. lack some key apoptotic machinery like caspases. Some molecular markers (e.g. PS exposure and DNA fragmentation) are observed, but the complete pathway is not conserved.

Regulated cell death (RCD) in *Leishmania* parasites serves adaptive and altruistic roles, benefiting the population’s survival and promoting disease transmission (Lee et al., 2002). In the sand fly vector, *Leishmania* parasites undergo RCD to regulate their population (Basmaciyan and Casanova, 2019). Non-infectious promastigotes that fail to differentiate into the infectious metacyclic stage die *via* RCD. This process ensures that essential nutrients, limited in the sand fly gut, are conserved for the infectious metacyclic forms, promoting their survival (Debrabant and Nakhasi, 2003). This is described as an altruistic strategy because sacrificing some parasites benefits the survival and propagation of the fittest.

Moreover, RCD controls parasite density within the host to prevent hyperparasitism, where excessive parasite burden could prematurely kill the host, thereby hindering transmission to a new host (Bruchhaus et al., 2007; Lüder et al., 2010). Additionally, macrophages that engulf apoptotic *Leishmania* cells secrete anti-inflammatory chemicals (e.g. TGF-β, IL-10, and lipoxin A4), which suppress the immune response. These macrophages also inhibit the synthesis of pro-inflammatory chemicals (e.g. leukotriene B4 and TNF-α), weakening the host’s immune defense. Consequently, this immune suppression benefits the surviving parasites (Van Zandbergen et al., 2006; Wanderley et al., 2009).

Furthermore, phagocytosis of apoptotic parasites by macrophages decreases their ability to present parasite antigens, further dampening the immune response (Zangger et al., 2002). Apoptotic parasites also trigger autophagy in the host, which reduces T-cell proliferation and aids in parasite survival within the host (Crauwels et al., 2015).

## 6 Apoptotic mimicry

In amastigotes, apoptotic mimicry refers to their ability to display PS exposure without undergoing cell death. By presenting PS exposure, amastigotes are identified and engulfed, mimicking the anti-inflammatory features of apoptotic cells (Fig. 2B). Phagocytosis of apoptotic *Leishmania* cells by macrophages induces anti-inflammatory responses (e.g. IL-10 and TGF-β), suppresses pro-inflammatory cytokines (e.g. TNF-α), and inhibits antigen presentation, aiding parasite survival (Fig. 2B) (Maspi et al., 2016; dos Santos Meira and Gedamu, 2019; Pacheco-Fernandez et al., 2021; Gurjar et al., 2022; Almeida et al., 2023).

Researchers discovered that amastigotes exhibit PS exposure and evade cell death through a mechanism driven by non-polarized macrophage activation, characterized by simultaneous activation of arginase I and iNOS, mediated by anti-*Leishmania* CD4^+^ T cells. It is suggested that the non-polarized response triggers PS exposure in reaction to nitric oxide-induced stress, while enhanced polyamine synthesis promotes parasite survival (Fig. 2C). Infections with *Leishmania amazonensis* typically elicit a weak host immune response that does not conform to the traditional Th1/Th2 dichotomy in some *Leishmania major* infections. CD4^+^ T cells in *L. amazonensis*-infected mice produce a combination of Th1 cytokines (TNF and IFN-γ), Th2 cytokines IL-5, (IL-4 and IL-13), and regulatory cytokines (TGF-β and IL-10) (Heinzel et al., 1989; Ji et al., 2002, 2003).

## 7 Apoptotic death

The behavior of promastigotes differs from that of amastigotes. During the transition from the logarithmic to the stationary phase in *L. major* cultures, the proportion of promastigotes displaying PS on their surface increases (Tripathi and Gupta, 2003). Similarly, a subset of promastigotes isolated from the midgut of the sand fly *Phlebotomus dubosqui* has been identified as PS-exposing based on their ability to bind Annexin-V, indicating apoptotic death in these parasites. Interestingly, in a model of footpad swelling, the presence of apoptotic promastigotes within the inoculum of non-apoptotic, viable parasites was essential for disease progression (Van Zandbergen et al., 2006) (Fig. 2A). These apoptotic promastigotes also played a role in deactivating polymorphonuclear cells, enhancing the intracellular survival of viable, infective parasites (El-Hani et al., 2012). Van Zandbergen et al. (2006) proposed that the apoptotic parasites support the survival of non-apoptotic ones, representing altruistic behavior within the *Leishmania* population (Van Zandbergen et al., 2006). A comparable phenomenon has been observed in *L. amazonensis* promastigotes (Wanderley et al., 2009). During normal metacyclogenesis and within the gut of *Lutzomyia longipalpis*, a subset of metacyclic parasites undergoes apoptosis. Apoptotic promastigotes were detected only at the anterior midgut-to-foregut boundary of the sand fly vector, indicating that this form of cell death is not random but rather a component of the differentiation process *in vivo*. Both PS-exposing apoptotic and non-exposing viable parasites must be present for effective macrophage infection (Fig. 2A: a,b). In these scenarios, the viable non-exposed parasites act as the infective agents, while the apoptotic PS-exposing subpopulation suppresses the inflammatory response of macrophages.

## 8 *Leishmania* apoptosis phenotype

In *Leishmania* spp., various triggers cause apoptosis-like features comparable to those in mammals. Stimuli include reactive oxygen species (ROS) (e.g. hydrogen peroxide and nitric oxide), heat shock, antimicrobial agents (e.g. novobiocin, amphotericin B, and miltefosine), anticancer drugs (e.g. miltefosine and doxorubicin), plant-derived compounds (e.g. withaferin A, camptothecin, and *Aloe vera* extracts), and lipids like edelfosine (Fig. 2C) (Jiménez-Ruiz et al., 2010; Smirlis et al., 2010). These agents maintain the integrity of the plasma membrane while causing cell rounding, chromatin shrinkage, nuclear fragmentation, and mitochondrial alterations (Padmanabhan et al., 2012).

It is still difficult to fully understand the molecular mechanisms behind *Leishmania* cell death because very little is known about its essential proteins. Unlike higher organisms, *Leishmania* cells undergoing apoptosis do not have apoptotic bodies or membrane blebbing (Jiménez-Ruiz et al., 2010). Their small size complicates the visualization of chromatin condensation *via* fluorescence microscopy. Annexin V binds phosphatidylserine in metazoans and is not a reliable apoptosis marker in *Leishmania* spp., as it binds other phospholipid classes (Weingärtner et al., 2012). However, Calcein, combined with propidium iodide, has emerged as a useful marker to distinguish cell states (Basmaciyan et al., 2017). Furthermore, *Leishmania* spp. lack caspases; therefore, other markers are needed to characterize apoptosis (Proto et al., 2013).

Based on standards for yeasts (Carmona-Gutierrez et al., 2018), Basmaciyan et al. (2018a) developed criteria for identifying apoptosis in *Leishmania* spp. First, growth curves and propidium iodide (PI) staining confirm cell death by showing a loss of viability and plasma membrane integrity. At least two signs, such as cell rounding, DNA fragmentation, shrinkage, mitochondrial depolarization, or membrane changes, are then used to detect apoptosis. The TUNEL assay is the most effective way to assess DNA fragmentation. Since autophagic cells lack these characteristics, these markers help differentiate between apoptosis and autophagy 32 in conjunction with Annexin V staining. In addition, apoptotic kinetics should track transitions from early to late apoptosis to distinguish it from necrosis (Basmaciyan and Casanova, 2019).

## 9 Proteins involved in *Leishmania* spp. apoptosis

### 9.1 Calpains

**Fig. 3:**
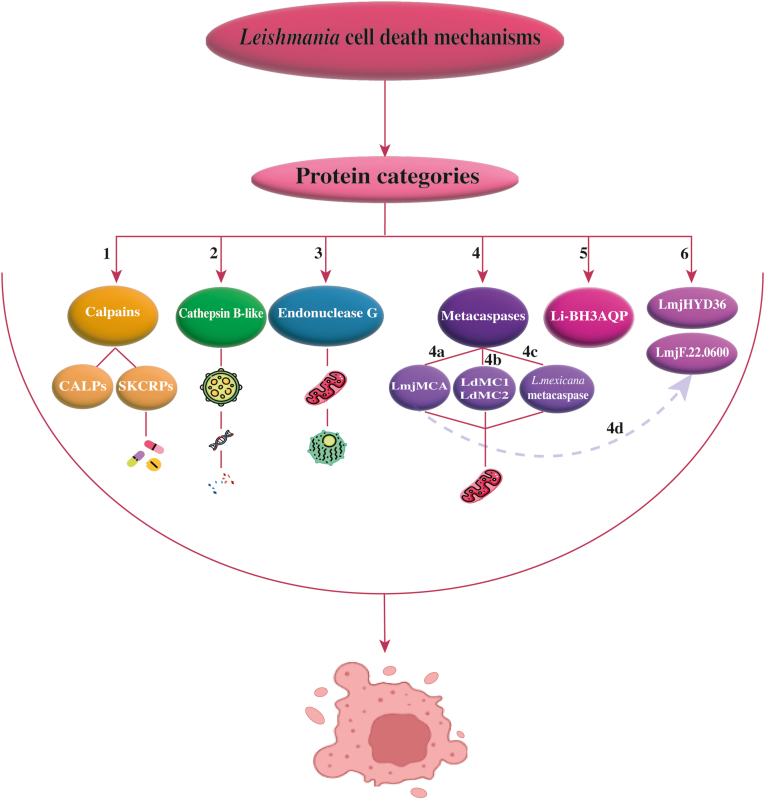
Proteins involved in *Leishmania* cell death mechanisms: calpains including CALPs and SKCRPs (**1**); cathepsin B-like protein (CPC) (**2**); endonuclease G (EndoG) (**3**); metacaspases including multiple subnodes (**4**) such as LmjMCA (**4a**), LdMC1 and LdMC2 (**4b**), and *L. mexicana* metacaspase (**4c**); Li-BH3AQP (**5**); and LmjHYD36 and LmjF.22.0600 (**6**). CALPs are associated with apoptosis but not consistent across stimuli. SKCRPs modulate apoptosis based on drug types, e.g. antimonials *vs* miltefosine. CPC, with its lysosomal origin, has a role in DNA degradation upon stress. EndoG, with its mitochondrial origin, participates in nuclear translocation during apoptosis and interacts with FEN-1 and TatD for DNA degradation. All metacaspases have a mitochondrial origin, and LmjMCA functions like trypsin and regulates LmjF.22.0600. *Leishmania mexicana* metacaspase has a dual role in apoptosis and population control. LdMC1 activates acidocalcisomes and the apoptotic process. LmjHYD36 has a catalytic triad but an uncertain DNA-binding role. LmjF.22.0600 interacts with LmjMCA and its compensatory apoptotic regulation (**4d**). The expression of LmjF.22.0600 depends on the presence of LmjMCA, indicating that LmjMCA regulates the expression of LmjF.22.0600.

Calpains are enzymes that require calcium and are present in mammals and *Leishmania* spp. (Dey et al., 2006; Ennes-Vidal et al., 2017). Unlike mammals, *Leishmania* spp. have several calpain-like proteins. These proteins can be divided into two categories: calpain-like proteins (CALPs) and small kinetoplastid calpain-related proteins (SKCRPs) (Fig. 3(1)) (Ersfeld et al., 2005).

While some studies suggest that calpain-like proteins are involved in *Leishmania* cell death, conflicting evidence exists regarding their role in apoptosis induced by various stimuli (Marinho et al., 2014; Ennes-Vidal et al., 2019). For example, calpain inhibitors do not affect *Leishmania* cell death caused by amphotericin B. Still, they inhibit apoptosis induced by nitric oxide (Holzmuller et al., 2002). A calpain inhibitor does not stop cell shrinkage but reduces miltefosine-induced DNA breakage (Paris et al., 2004).

SKCRP14.1, present in an antimonial-resistant strain, promotes antimonial-induced apoptosis while protecting against miltefosine-induced apoptosis (Vergnes et al., 2007). Additionally, the interaction between LmCALP27.2 and the C-terminal portion of LmjMCA, another protein implicated in *Leishmania* apoptosis, further suggests a possible functional relationship between these two proteins (Basmaciyan and Casanova, 2019).

### 9.2 Cathepsin B-like protein (CPC)

Cysteine proteinase C (CPC) is a type of protein classified as a cathepsin B-like protein (Barman et al., 2018). Cathepsins are enzymes that degrade proteins and are commonly found in lysosomes. This enzyme is expressed in *L. major* and can bind to the pan-caspase inhibitor Z-VAD-FMK. This interaction suggests that CPC plays a role in apoptosis, potentially mimicking or replacing caspase functions in *Leishmania* (El-Fadili et al., 2010). When *Leishmania* cells are exposed to stressors, CPC is discharged from lysosomes into the cytosol, directly triggering the processes leading to apoptosis in *Leishmania* (Fig. 3(2)) (El-Fadili et al., 2010).

### 9.3 Endonuclease G (EndoG)

*Leishmania donovani* and *L. infantum* have been used to study EndoG which acts as a nuclease and performs similar tasks to those seen in other species (Rico et al., 2014). Three conserved catalytic amino acids, arginine (R), glycine (G), and histidine (H), are necessary for EndoG nuclease activity (BoseDasgupta et al., 2008; Gannavaram et al., 2008; Rico et al., 2009). Under normal situations, EndoG is found in the mitochondria of both promastigote and axenic amastigote forms of the parasite. An amino acid sequence within the mitochondria suppresses EndoG nuclease activity (Oliva et al., 2017). However, in response to apoptotic stimuli, such as hydrogen peroxide or the drug edelfosine, EndoG translocates from the mitochondrion to the nucleus (Fig. 3(3)). In the nucleus, it forms complexes with Flap endonuclease-1 (FEN-1) and TatD nuclease. These complexes collaboratively degrade DNA through exonuclease and endonuclease activities (BoseDasgupta et al., 2008).

### 9.4 Metacaspases

*Leishmania* spp. possess cysteine peptidases called metacaspases. These enzymes are structurally similar to caspases but differ in substrate specificity. While caspases cleave at aspartate residues, metacaspases cleave at arginine or lysine residues (González et al., 2007; Vercammen et al., 2007; Meslin et al., 2011). They contain conserved catalytic amino acids (histidine and cysteine) that drive their enzymatic activity (Tsiatsiani et al., 2011; Zalila et al., 2011). Metacaspases are involved in apoptosis, triggered by various stresses or drugs, but their functions extend beyond cell death to processes such as autophagy and proliferation regulation (Casanova et al., 2015; Vandana et al., 2019). Their activity, processing, and localization variations highlight their varied functions in parasite biology and survival methods.

Different types of *L. major* (promastigotes and amastigotes) express the metacaspase LmjMCA (Ambit et al., 2008). Although it can move to the mitochondrion, its primary location is in the cytoplasm. While LmjMCA lacks caspase-like activity, it exhibits trypsin-like activity dependent on its catalytic dyad (His147/Cys202) (Martin et al., 2014). During apoptosis, stimuli such as oxidative stress, heat shock, or drugs (e.g. miltefosine and curcumin) induce LmjMCA auto-processing, releasing a catalytic domain that executes apoptosis by cleaving substrates (Fig. 3(4a)) (Zalila et al., 2011).

Two metacaspases, LdMC1 and LdMC2, have been identified in *L. donovani*; however, the status of LdMC2 remains uncertain due to inconsistent evidence (Lee et al., 2007). LdMC1 is expressed in both amastigotes and promastigotes and is localized in acidocalcisomes, where the acidic pH inhibits its activity. LdMC1 has trypsin-like activity, and under apoptotic conditions (e.g. H_2_O_2_ exposure), LdMC1 is released from acidocalcisomes to induce apoptosis (Lee et al., 2007). LdMC1 may also play a role in parasite survival, although further research is needed to confirm this claim (Fig. 3(4b)) (Raina and Kaur, 2012).

The single metacaspase in *L. mexicana* is primarily cytoplasmic. It contributes to apoptosis induced by miltefosine but does not affect susceptibility to other stresses (e.g. oxidative stress). Additionally, this metacaspase inhibits the growth of intracellular amastigotes, suggesting a dual role in regulating cell death and population control (Fig. 3(4c)) (Castanys-Muñoz et al., 2012).

### 9.5 Li-BH3AQP

The Li-BH3AQP protein is an aquaporin in *L. infantum*. It has a Bcl-2 homology (BH3) domain, a part of proteins in the Bcl-2 family (Fig. 3(5)) (Genes et al., 2016). Li-BH3AQP can attach to the anti-apoptotic molecule Bcl-XL in mammalian cells, decreasing cell survival. This protein has been reported to be the first non-enzymatic molecule related to the death of *Leishmania* cells. This protein, mostly found near the nucleus in *L. infantum*, has pro-death properties. When exposed to pro-apoptotic stimuli such as staurosporine and antimycin A, this activity is associated with certain residues in the BH3 domain that cause DNA fragmentation and reduce cell viability. However, Li-BH3AQP exhibits pro-survival activity under hypotonic stress or nutrient deficiency conditions (Genes et al., 2016).

### 9.6 LmjHYD36 and Lmj.22.0600

LmjHYD36 and LmjF.22.0600 are two conserved proteins among *Leishmania* spp. In *L.* *major*, they function as cell death effectors (Basmaciyan et al., 2019a, Basmaciyan et al., 2019b). LmjHYD36 contains the catalytic triad of cysteine, histidine, and aspartic acid, typical of α/β-hydrolases. *Leishmania* species 55 share a high degree of conservation in this protein. Despite being identified in the TriTryp database as a potential endonuclease, its absence of the structural characteristics necessary for DNA binding and its lack of obvious nuclease activity raise doubts about its endonuclease function (Fig. 3(6)) (Basmaciyan et al., 2019b).

LmjF.22.0600, as an acetyltransferase, is upregulated during exposure to various pro-apoptotic drugs (Basmaciyan et al., 2019a), such as curcumin and miltefosine. In contrast, when the *LmjF.22.0600* gene is deleted, this does not significantly affect cell death in *L. major*. This observation proposes that another protein compensates for its absence, ensuring the apoptotic pathway continues. In this regard, LmjF.22.0600 and LmjMCA function within the same apoptotic pathway. The expression of LmjF.22.0600 depends on the presence of LmjMCA, indicating that LmjMCA regulates the expression of LmjF.22.0600 (Fig. 3(4d)) (Basmaciyan et al., 2018a).

## 10 Advantages of host cell apoptosis in *Leishmania* spp. pathogenesis

**Fig. 4:**
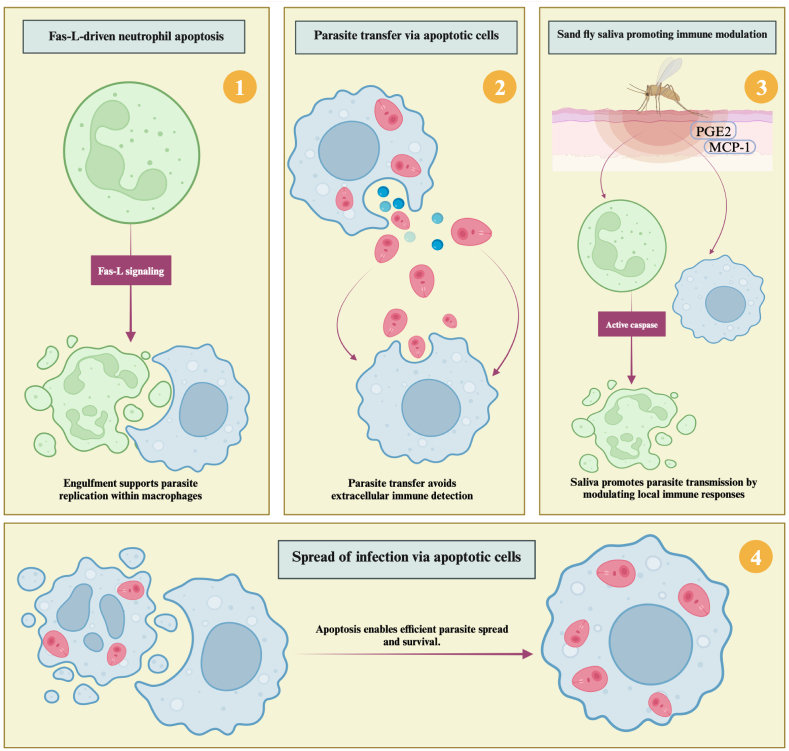
The role of apoptosis in supporting *Leishmania* infection. (**1**) Fas-L-mediated apoptosis in neutrophils and apoptosis in neutrophils engulfed by macrophages can enhance parasite replication. (**2**) When amastigotes transfer from an apoptotic macrophage to a healthy macrophage through zeiotic structures (membrane-bound vesicles), the parasite is protected from exposure to extracellular factors. (**3**) By biting a host, sand flies release saliva into the skin, leading neutrophils near the bite site to undergo apoptosis, caspase activation, and reduced ROS production. In addition, saliva increases prostaglandin E2 (PGE2) and MCP-1, which attract macrophages and facilitate parasite transmission. (**4**) Apoptotic cells attract healthy macrophages, which phagocytose apoptotic, infected macrophages, thus allowing amastigotes to infect new cells, and these apoptotic processes enhance parasite survival and virulence. Created in BioRender (https://BioRender.com/x23q402).

Research has revealed that promoting apoptosis might support *Leishmania* spp. infection (Fig. 4). Fas-L-dependent apoptosis of neutrophils has been found to play a crucial role in maintaining *L. major* in BALB/c mice genetically susceptible to the disease. This mechanism enhances parasite replication in neutrophils, which apoptotic local macrophages recruit to the infection site (Fig. 4(1)) (Ribeiro-Gomes et al., 2005).

According to Baars et al. (2023), *L. major* reduces the possibility that the host’s immune system will discover and eliminate it by inducing apoptosis to transfer between host cells without exposure to the extracellular environment (Fig. 4(2)) (Baars et al., 2023). Likewise, *L. amazonensis* amastigotes have been shown to move from infected cells to newly recruited monocytes without being completely exposed to the environment (Real et al., 2014). These authors found that *L. amazonensis* amastigotes were transferred from one cell to another when the donor macrophage signaled impending apoptosis. During this process, the amastigotes are expelled from the macrophage inside small, membrane-bound structures called zeiotic structures, a feature of apoptosis. Nearby macrophages subsequently collect the released amastigotes, keeping them alive and functional (Fig. 4(2)).

According to Prates et al. (2011), the saliva of the sand fly *L. longipalpis* changes host immunological responses to provide an environment favorable to the survival and growth of *L. chagasi* and *L. infantum* within neutrophils. In particular, the saliva stimulates neutrophil death through caspase and FasL. Saliva inhibits the production of ROS, which are normally involved in the removal of pathogens, during this process. There is also an increase in the production of the anti-inflammatory chemical prostaglandin E2 (PGE2). Additionally, infected neutrophils generate a lot of monocyte chemoattractant protein-1 (MCP-1), chemotactically attracting macrophages to the infection site. The parasites may be able to continue their life cycle by infecting macrophages more easily due to this recruitment (Fig. 4(3)). As a result, neutrophils carry more parasites, which helps the illness spread and last longer (Prates et al., 2011).

The spread of *L. amazonensis* between cells is also facilitated by apoptotic cells. More specifically, healthy macrophages ingest apoptotic, infected macrophages, potentially allowing the amastigotes to move into the new host cells. Furthermore, because *L. amazonensis* amastigotes can infect new cells by causing apoptosis, they are more virulent than amastigotes of *L. guyanensis* in BALB/c and C57BL/6 mice. This process includes DNA fragmentation, hypodiploidy, and phosphatidylserine (PS) exposure on the cell surface. Caspases-3, -8, and -9 are the enzymes that cause this apoptosis. In contrast to *L. amazonensis*, *L. guyanensis* does not cause infected macrophages to undergo apoptosis. Instead, necrosis kills these macrophages (DaMata et al., 2015) (Fig. 4(4)).

## 11 Evasion strategies of *Leishmania* spp. from host cell apoptosis

**Fig. 5:**
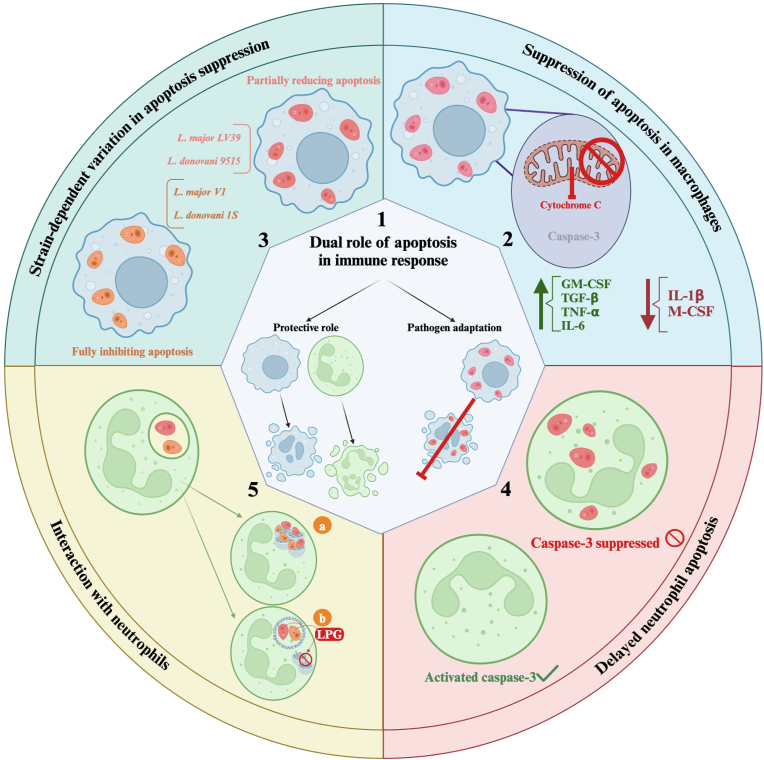
The modulation of apoptosis during *Leishmania* infections. (**1**) The dual role of apoptosis in the immune response (both a protective and pathogen adaptation role), is illustrated. *Leishmania* manipulates apoptosis in host cells to promote its survival as a pathogen adaptation by removing infected cells and attracting immune cells to clear the pathogen. (**2**) Suppression of apoptosis in macrophages: in a macrophage infected with *L. donovani*, GM-CSF, TGF-β, TNF-α, and IL-6 are upregulated, and IL-1β and M-CSF are downregulated. (**3**) Strain-dependent variation in apoptosis suppression: strains fully inhibiting apoptosis (e.g. *L. major* V1, Spock, IR173 and *L. donovani* 1S) and strains partially reducing apoptosis (e.g. *L. major* LV39, NIH S and *L. donovani* 9515, Mongi). (**4**) Delayed neutrophil apoptosis: neutrophils without Caspase-3 are infected with *Leishmania*, but neutrophils with activated Caspase-3 are not. (**5**) Interaction with neutrophils: *L. major* and *L. donovani* promastigotes are being phagocytosed by neutrophils. The neutrophils have two compartments: the lysosome-fused compartment that destroys parasites and the ER-like compartment that shields parasites from degradation (**a**), as well as the LPG compartment that prevents lysosome fusion (**b**). Created in BioRender (https://BioRender.com/x23q402).

An essential part of the immune system’s reaction to infections is apoptosis. In particular, macrophages can initiate apoptosis as a protective strategy. Apoptosis has two functions. First, removing infected cells eliminates the environment that allows microorganisms to survive (Fernandes and Zamboni, 2024). Secondly, it facilitates the elimination of pathogens by directing other immune cells to engulf and remove the microorganisms (Fig. 5(1)) (Gupta et al., 2013). Pathogens and hosts have impacted each other’s evolutionary adaptations over time. Numerous mechanisms that control apoptosis during infection have been developed due to this dynamic interaction between hosts and *Leishmania* spp. (Shio et al., 2012; Wenzel et al., 2012; Basmaciyan et al., 2018b; Solano-Gálvez et al., 2021). For example, certain pathogens may adapt to increase their lifespan to prevent apoptosis (Chow et al., 2016).

Apoptosis in bone marrow-derived macrophages (BMDMs) can be suppressed by infection with *L. donovani* or exposure to its lipophosphoglycan (LPG), a crucial surface molecule. This usually happens when M-CSF, a growth factor necessary for macrophage survival and function, is deficient. This infection upregulates explicitly the expression of granulocyte-macrophage colony-stimulating factor (GM-CSF), TGF-β, TNF-α, and IL-6 while downregulating the expression of M-CSF and IL-1 beta (Fig. 5(2)). This gene expression pattern suggests that *L. donovani* promotes its survival and persistence while potentially hindering the host’s capacity to mount a successful immune response (Moore and Matlashewski, 1994).

Similarly, in the U-937 human monocytic cell line, components produced from *L. infantum* prevent actinomycin D-induced death (Lisi et al., 2005). Enhancing the parasite’s survival probably involves blocking signaling pathways regulated by protein kinase C (PKC). LPG may assist the parasite in avoiding host immune responses by blocking its routes and prolonging its survival in host cells.

Akarid et al. (2004) revealed that infection with *L. major* protects murine macrophages from apoptosis caused by M-CSF deprivation and staurosporine treatment. The protection from cell death is associated with inhibiting the activation of effector caspases and the release of cytochrome *c* while bypassing typical survival pathways like NF-κB (Fig. 5(2)). Moreover, they observed that the ability of *L. major* to prevent apoptosis occurs in both resistant C57BL/6 mice and susceptible BALB/c, irrespective of genetic differences (Akarid et al., 2004).

Donovan et al. (2009) showed that different strains of *Leishmania* have varying abilities to inhibit apoptosis. Differences may influence this ability in the molecular composition of the parasite surface structures, which interact with the host immune system. In this regard, among the tested strains of *L. major*, V1, Spock, and IR173 fully inhibited apoptosis in infected macrophages. In contrast, strains LV39 and NIH S reduced apoptosis but were less effective than the first three strains. A similar strain-dependent effect is observed in *L. donovani*. For example, strain 1S completely inhibited apoptosis, while strains 9515 and Mongi only partially reduced apoptosis (Fig. 5(3)) (Donovan et al., 2009).

Neutrophils are among the first host cells encountered by *Leishmania* spp. during infection (Müller et al., 2001). Polymorphonuclear neutrophil granulocytes (PMNs) can take in *Leishmania* promastigotes. However, these immune cells are not usually considered suitable parasite hosts. The phagocytosis of live *L. major* by neutrophils delays neutrophil apoptosis both *in vitro* and *in vivo* by inhibiting Caspase-3 activation (Aga et al., 2002). Nevertheless, the supernatants could not replicate this ability of the parasite. This suggests that the antiapoptotic effect requires direct interaction between the parasites and the PMNs rather than being mediated by substances secreted into the surrounding fluid (Fig. 5(4)).

Likewise, *L. donovani* prolongs the lifespan of neutrophils. Generally, *L. donovani* promastigotes are directed into two distinct compartments within neutrophils. In one compartment type, lysosomes form highly destructive environments where the parasites are quickly broken down (Fig. 5(5a)). In the other type of compartment, lysosomes are not involved. Instead, the compartment exhibits characteristics resembling the endoplasmic reticulum (ER). In these ER-like compartments, the parasites are shielded from degradation. During neutrophil infection with *L. donovani*, the parasite LPG prevents lysosome fusion, enabling the parasites to survive within the ER-like compartments (Fig. 5(5b)) (Gueirard et al., 2008).

## 12 *Leishmania* spp. molecular strategies for disrupting apoptotic signaling pathways

**Fig. 6:**
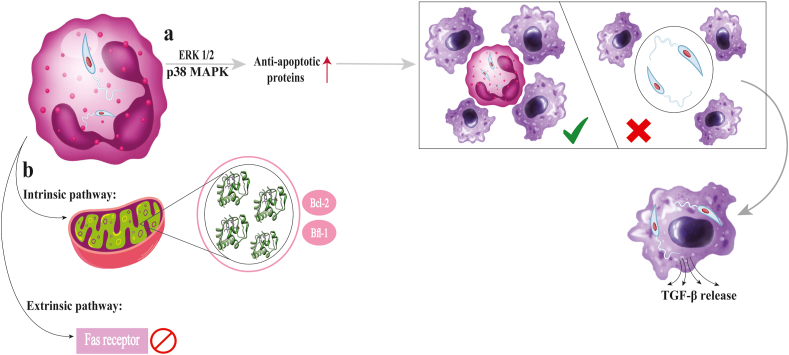
*Leishmania* as a destructive microscopic parasite through apoptosis invades neutrophils and macrophages, hijacking their natural death mechanisms to ensure its own survival (**a**). Normally, infected cells would self-destruct through apoptosis, but it interferes with both intrinsic and extrinsic pathways, blocking cytochrome *c* release from mitochondria and preventing Fas-mediated apoptosis (**b**). This manipulation extends the neutrophil lifespan, allowing the parasite to hide inside and eventually be engulfed by macrophages without triggering an immune response. Once inside, *Leishmania* thrives, activating the PI3K/Akt pathway to block cell death signals while suppressing immune defenses with TGF-β.

Different *Leishmania* spp. may disrupt apoptotic processes in the host cells and trigger various signaling pathways. *Leishmania major* affects both intrinsic and extrinsic apoptosis (Fig. 6(a,b)) (Fernandes and Zamboni, 2024). When neutrophils are infected with *L. major*, the parasite manipulates the cells’ internal signaling pathways (p38 MAPK and ERK1/2) to increase the expression of anti-apoptotic proteins (Bfl-1 and Bcl-2) (Fig. 6(a,b)). This efficiently suppresses apoptosis and prolongs parasite survival within the host cells by preventing the secretion of cytochrome *c* from mitochondria. Additionally, *L. major* limits the activation of extrinsic apoptosis by decreasing the expression of FAS on neutrophils (Sarkar et al., 2013).

MAP kinases trigger apoptosis, and their decreased activity may impede this process. Vázquez-López et al. (2015) reported that amastigotes of *L. mexicana* remarkably reduced the activity of two MAP kinases, JNK and p38. This suppression led to a considerable reduction in DNA fragmentation in dendritic cells. Interestingly, amastigotes of *L. mexicana* were able to trigger the AKT and PI3K signaling pathways, which are known to promote cell survival and oppose apoptosis, even while they suppressed MAP kinase activity (Fig. 6(a)) (Vázquez-López et al., 2015).

According to a study by Ruhland et al. (2007), three important host signaling pathways, p38 mitogen-activated protein kinase (MAPK), nuclear factor kappa B (NFκB), and phosphoinositide 3-kinase/Akt (PI3K/Akt), are activated by the promastigote form of three *Leishmania* spp. (*L. major*, *L. pifanoi*, and *L. amazonensis*). However, the resistance to apoptosis caused by the parasite was not significantly affected by blocking NFκB signaling with wedelolactone or p38 MAPK signaling with the inhibitor SB202190. According to this research, PI3K/Akt signaling is more important than the other pathways in helping infected cells avoid apoptosis (Ruhland et al., 2007).

*Leishmania amazonensis* manipulates macrophages to suppress all major RCD pathways. Macrophages infected with amastigotes of *L. amazonensis* resist apoptosis from intrinsic (CSF-1 deprivation) and extrinsic (actinomycin D treatment) pathways. Infection also inhibited LPS/ATP-induced pyroptosis, significantly extending macrophage viability to over 50 days (Lecoeur et al., 2022).

*Leishmania infantum* prevents apoptosis in a macrophage-like cell line (U-937 cells) by upregulating the production of anti-apoptotic proteins Bcl-2 and cIAP1/2 (cellular inhibitors of apoptosis proteins). Cianciulli et al. (2018) also discovered that when U-937 cells were infected with *L. infantum* and treated with the Bcl-2 inhibitor ABT-737, the level of apoptosis increased substantially. According to this research, *L. infantum* uses the Bcl-2 protein to aid infected macrophages in avoiding apoptosis, and blocking Bcl-2 can suppress this impact (Cianciulli et al., 2018).

*Leishmania donovani* has evolved several strategies to modify macrophage immunological and metabolic processes to assist the parasite in living inside them. According to Giri et al. (2016), *L. donovani* prevents infected macrophages from dying by upregulating the host’s MCL-1 protein. By activating CREB (cAMP-response element-binding protein) and promoting the trafficking of MCL-1 to mitochondria *via* TOM70, the parasite accomplishes this process*.* Once in the mitochondria, MCL-1 blocks pro-apoptotic proteins like BAK. Silencing MCL-1 disrupts this protective mechanism, making macrophages and splenocytes more susceptible to apoptosis, consequently reducing parasite load (Giri et al., 2016).

Pandey et al. (2016) investigated the role of the host protein Bcl-2 during *L. donovani* infection. They demonstrated that the parasite suppresses nitric oxide synthesis in macrophages and causes a two-fold rise in Bcl-2 expression during infection, contributing to the parasite’s intracellular survival. Interestingly, the parasite’s capacity to persist inside the host cells was diminished when Bcl-2 levels were decreased by siRNA or Bcl-2 inhibitors. The parasite induces Bcl-2 expression through a Th2 cytokine (IL-13), which activates the STAT-3 transcription factor. Moreover, the upregulation of Bcl-2 is linked to Toll-like receptor (TLR)-2 signaling *via* the MEK-ERK pathway, which also affects how effectively host cells take up parasites (Pandey et al., 2016).

Gupta et al. (2016) demonstrated that *L. donovani* activates AKT to manipulate the GSK-3β/β-catenin/FOXO-1 axis, suppressing apoptosis and the immune response to ensure its survival inside macrophages. Specifically, infection activates AKT, which phosphorylates and deactivates GSK-3β, a protein that typically prevents β-catenin from entering the nucleus. Once GSK-3β is inhibited, β-catenin moves into the nucleus, stimulating anti-apoptotic signals to help infected macrophages survive. Furthermore, FOXO-1 is phosphorylated and inhibited by AKT activity, which stops it from entering the nucleus. This activity dampens inflammatory signals and further decreases apoptosis (Gupta et al., 2016).

During an immune response, macrophages usually produce ROS, such as H_2_O_2_, to induce apoptosis and help eliminate infections. However, *L. donovani* blocks this process, increasing the infected macrophages’ resistance to H_2_O_2_-induced apoptosis. Even though the infected macrophages produce ROS, Srivastav et al. (2014) showed that the downstream effects like the activation of caspase-3, caspase-7, and PARP cleavage, which trigger apoptosis, are greatly diminished. The upregulation of protective proteins, such as thioredoxin SOCS1 and SOCS3 (suppressors of cytokine signaling), allows this suppression (Srivastav et al., 2014).

AKT phosphorylation and its pro-survival effects are restored when PD-1 expression is decreased during *L. donovani* infection. Infected macrophages can withstand apoptosis because phosphorylated AKT suppresses the pro-apoptotic protein BAD. *Leishmania donovani* inhibits the activity of NFATc1 (nuclear factor of activated T-cells, cytoplasmic 1), actively decreasing PD-1 production in macrophages. Furthermore, *L. donovani* interferes with the interaction between SHP2 and PD-1, deactivating AKT, and enabling AKT to stay active and stop apoptosis (Roy et al., 2017).

## 13 Conclusions

*Leishmania* parasites display complex apoptotic-like machinery, which is crucial for their survival and interactions with the host. These critical pathways, distinct from mammalian apoptosis, offer a promising therapeutic potential. Despite the lack of approved vaccines, this strategy facilitates the development of drugs that selectively target *Leishmania*-specific apoptotic pathways, such as those involving metacaspases, mitochondrial dysfunction, and oxidative damage. Therefore, this approach has the potential to improve therapeutic efficacy while minimizing adverse effects in leishmaniasis therapeutic responses by exploiting parasite-specific vulnerabilities. Further challenges remain in future directions in this field, emphasizing the crucial need for a deeper understanding of the merits and mediators of underlying molecular mechanisms.

## CRediT authorship contribution statement

**Soheil Sadr:** Writing – original draft, Writing – review & editing, Formal analysis, Data curation, Validation, Conceptualization, Methodology. **Iraj Sharifi:** Project administration, Conceptualization, Writing – original draft, Writing – review & editing, Formal analysis, Methodology. **Solmaz Morovati:** Writing – review & editing, Methodology, Investigation, Formal analysis, Data curation, Validation. **Helia Sepahvand:** Conceptualization, Writing – review & editing, Project administration, Methodology, Investigation, Data curation, Funding acquisition. **Shakiba Nazemian:** Writing – review & editing, Formal analysis. **Mehdi Bamorovat:** Writing – original draft, Writing – review & editing, Formal analysis, Data curation. **Zahra Rezaeian:** Writing – review & editing, Formal analysis, Data curation. **Baharak Akhtardanesh:** Writing – original draft, Writing – review & editing, Formal analysis, Data curation.

## Ethical approval

Not applicable.

## Funding

No funding was received for this study.

## Declaration of competing interest

The authors declare that they have no known competing financial interests or personal relationships that could have appeared to influence the work reported in this paper.

## Data Availability

The data supporting the conclusions of this article are included within the article.
